# The Emerging Role and Promise of Circular RNAs in Obesity and Related Metabolic Disorders

**DOI:** 10.3390/cells9061473

**Published:** 2020-06-16

**Authors:** Mohamed Zaiou

**Affiliations:** 1 School of Pharmacy, The University of Lorraine, 7 Avenue de la Foret de Haye, CEDEX BP 90170, F-54500 Vandoeuvre-les-Nancy, France; mohamed.zaiou@univ-lorraine.fr; Tel.: +3303-7277-90-15; Fax: +3303-8368-23-01; 2 Institut Jean Lamour, UMR 7198, CNRS, The University of Lorraine, 2 allée André Guinier, BP 50840, 54011 Nancy, France

**Keywords:** circular RNAs (circRNAs), adipogenesis, epigenetics, cardiovascular diseases, microRNAs (miRNAs), insulin resistance, adipose tissue browning

## Abstract

Circular RNAs (circRNAs) are genome transcripts that are produced from back-splicing of specific regions of pre-mRNA. These single-stranded RNA molecules are widely expressed across diverse phyla and many of them are stable and evolutionary conserved between species. Growing evidence suggests that many circRNAs function as master regulators of gene expression by influencing both transcription and translation processes. Mechanistically, circRNAs are predicted to act as endogenous microRNA (miRNA) sponges, interact with functional RNA-binding proteins (RBPs), and associate with elements of the transcriptional machinery in the nucleus. Evidence is mounting that dysregulation of circRNAs is closely related to the occurrence of a range of diseases including cancer and metabolic diseases. Indeed, there are several reports implicating circRNAs in cardiovascular diseases (CVD), diabetes, hypertension, and atherosclerosis. However, there is very little research addressing the potential role of these RNA transcripts in the occurrence and development of obesity. Emerging data from in vitro and in vivo studies suggest that circRNAs are novel players in adipogenesis, white adipose browning, obesity, obesity-induced inflammation, and insulin resistance. This study explores the current state of knowledge on circRNAs regulating molecular processes associated with adipogenesis and obesity, highlights some of the challenges encountered while studying circRNAs and suggests some perspectives for future research directions in this exciting field of study.

## 1. Introduction

Whole-genome sequencing studies reveal that our genome comprises approximately 3 to 3.2 billion base pairs of DNA, but merely 1–2% of those pairs correspond to the annotated exons of protein-coding genes. The remaining 98–99% have been referred to as “junk DNA” or “dark matter” with no obvious function in the cell [1]. Currently, major scientific advances in the field that have occurred upon implementation of novel and sensitive molecular approaches to genome-wide transcriptome analysis [2], have totally changed the way we look at the vast “noncoding” regions of the human genome. What was once considered “junk DNA” because of its lack of function is now viewed as a treasure. It is becoming known from multiple studies that junk DNA can be almost entirely transcribed, generating a huge number of functional transcripts commonly referred to as noncoding RNAs (ncRNAs) although many of their functions remain enigmatic [3,4]. According to the biological functions of ncRNAs, they can be classified into infrastructural and regulatory types. Infrastructural ncRNAs include ribosomal RNAs (rRNAs), transfer RNAs (tRNAs), and small nuclear RNAs (snRNAs) and are typically expressed constitutively, whereas regulatory ncRNAs consist mainly of microRNAs (miRNAs), long noncoding RNAs (lncRNAs), circular RNAs (circRNAs), and piwi-interacting RNAs (piRNAs). Inconsistent with the “central dogma of biology” that describes the flow of genetic information from gene to mRNA and finally to protein, ncRNAs are generally not further translated into proteins [5]. Although they do not encode proteins, these RNA molecules can still be endowed with critical regulatory function and may help orchestrate a hidden layer of gene regulation networks [6]. As a matter of fact, circumstantial evidence revealed that ncRNAs can act as critical switches that fine-tune target gene expression [7,8]. From a clinical perspective, several transcriptomic studies have unveiled that a large number of ncRNAs can be altered in major diseases [9] clearly hinting towards the potential therapeutic application of these RNA molecules in disease therapy.

As stated before, there are different types of ncRNA transcriptomes with varied functions; among the most relevant are small ncRNAs such as miRNAs (18–24 nucleotides (nt.)), lncRNAs (>200 nt.), and circRNAs (median length is ~530 nt.). In recent years, considerable attention has focused on circRNAs, which are distinguished from their linear counterparts by the unique structure of covalently closed continuous loop lacking 5′ cap and 3′ poly-adenylated tails [10]. This feature makes circRNAs remarkably stable and resistant to degradation by exonuclease and RNases, therefore, they are proposed as new generation of predictive biomarkers and potential therapeutic targets for many diseases. Even though evidence of their biological relevance and clinical significance in human disease pathogenesis is continuously emerging, our current knowledge of circRNAs remains preliminary in metabolic diseases and obesity in particular. Additionally, to the very best of my knowledge, there is hardly any comprehensive coverage on the role of circRNAs in the pathogenesis of obesity. Hence, the next sections will discuss relevant literature in the field and highlight some of the key aspects of circRNAs and challenges ahead.

## 2. Landscape of circRNAs

### 2.1. Biogenesis of circRNAs

**Figure 1:**
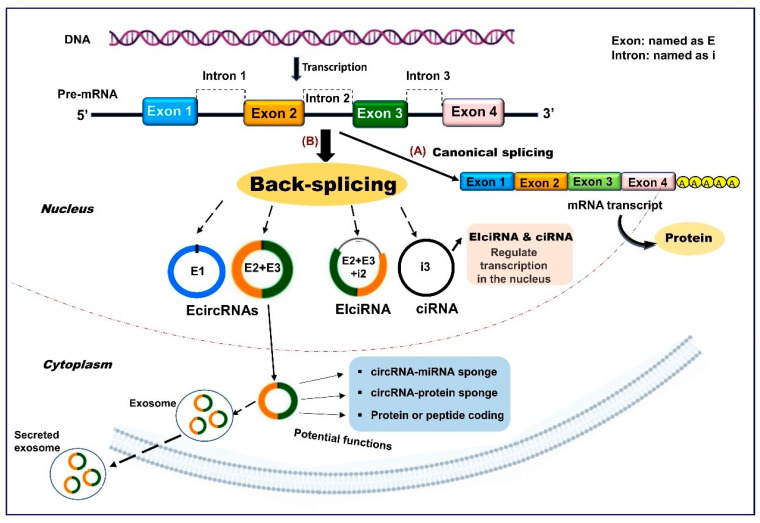
A simplified schematic representation of circular RNAs (circRNAs) biogenesis. (**A**) Pre-mRNA can undergo canonical splicing to generate a linear mRNA transcript that is subsequently translated into protein. (**B**) Pre-mRNA can also be spliced in the noncanonical manner “back-splicing”, wherein a downstream splice donor site is joined to an upstream splice acceptor site to produce circular RNA molecules. Three different types of circRNAs can arise from different genomic positions and combinations, including: (**1**) exonic circRNAs (EcircRNAs), (**2**) exon-intron circRNAs (EIciRNAs), and (**3**) circular intronic RNAs (ciRNAs). The formation mechanisms and potential functions of these circRNA types are discussed in the text.

Contrary to conventional splicing forming a linear RNA, circRNAs are generally derived from precursor mRNA back-splicing, a molecular event in which a downstream 5′ splice site is joined to a downstream 3′ splice site to form a covalently closed circular structure [11]. Even though the specific and detailed mechanisms mediating circRNAs biogenesis are not fully understood, several models have been proposed, including direct back-splicing with *Arthrobacter luteus* (ALU) elements and inverted repeats complementation, lariat-driven circularization (exon skipping), and RNA-binding-protein (RBP) mediated models [12]. Based on their sequences, circRNAs can be generally classified into three categories: i) exonic circRNAs (EcircRNAs), which are generated only from the exon regions, account for most (over 80%) known circRNAs, and are found mainly in the cytoplasm; ii) circRNAs derived from lariat introns (ciRNAs) predominantly located in nuclei; and iii) circRNAs derived from exons with retained introns (EIciRNAs) and can be mainly found in nuclei [10,11,13] (Figure 1). Previous analysis of the number of circRNAs from their host genes revealed that one gene could produce multiple circRNA isoforms, with nearly 50% of the host genes expressing one circRNA at significantly higher levels [14]. Concerning circRNAs turnover, exosomes have been proposed as one of the mechanisms by which these transcripts might be cleared from the cell [15], but further studies are required to explore the regulatory factors involved in the control of circRNAs metabolic turnover.

### 2.2. Properties of circRNAs

According to the published literature, circRNAs share several features. They are highly stable in cells due to their unique structure, and with most species, the average half-life period of these molecules is about 48 h which is much longer than that of mRNAs (10 h) [16]. However, this may not be the case for exonic circRNAs in serum, presumably due to circulating RNA endonucleases. circRNAs are evolutionarily conserved among species, diverse, often show tissue or development stage-specific expression patterns and have determined subcellular localization [16,17,18]. The abundance of circRNAs relative to their linear RNA counterparts varies between cell types [19]. They are abundant in exosomes and often found in extracellular fluid (like saliva, blood, and urine) with an expression level ten times higher than that of linear mRNAs [20]. Their size ranges from hundreds to thousands of nucleotides, while most of these RNA species in mammals and plants are hundreds of nucleotides [16,21] and usually contain between one and five exons [18].

### 2.3. Biological Functions of circRNAs

#### 2.3.1. circRNAs Can Function as miRNA Sponges

Although the exact function of most circRNAs is still ambiguous, several studies have indicated that circRNAs may control multiple biological processes via a variety of mechanisms. They can serve as miRNA sponges, which in turn influence target mRNA translation [18,22]. In other words, circRNAs that contain miRNA recognition elements (MREs) can interact with miRNAs by stable complementary base pairing [23,24] to inhibit their activity, and thus, effectively alter their role in the posttranscriptional regulation of target gene expression. To cite a few examples, the cerebellar degeneration-related antigen 1-antisense circRNA (CDR1as, also known as ciRS-7), which is highly expressed in the mammalian brain and upregulated during neuronal development [25], contains up to 74 binding sites for the miR-7 and can act as a decoy or sponge for this miRNA [18]. In human cells, knockdown of CDR1as expression suppresses miR-7 expression and affects insulin secretion, cell proliferation and the pathobiology of myocardial infarction [26,27,28]. Likewise, sex determining region Y (SRY)-derived circRNA (circSRY), which is expressed in murine testis and harbors 16 miR-138 conserved binding sites, has also been shown to act as a miRNA sponge [23,24] resulting in gene expression dysregulation.

#### 2.3.2. circRNAs Can Function as Transcription Regulators

In addition to circRNAs found in the cytoplasm compartment, a fraction can accumulate inside the nucleus (ciRNAs and EIciRNAs) where they control gene expression at the transcriptional level. These intron-retaining circRNAs can interact with upstream promoters, RNA polymerase II (Pol II), and other proteins of the transcription machinery to regulate their parental gene expression in some circumstances [29,30,31]. For example, the ciRNAs, ci-ANKRD52 and ci-SIRT-7, are able to accumulate at transcription sites and enhance their parental genes expression, ankyrin repeat domain 52 (*ANKRD52)* and sirtuin 7 (*RIRT7)* respectively, through interaction with Pol II elongation complex [30,32]. circEIF3J and circPAIP2, two exon-intron circRNAs, are an additional example of circRNAs exclusively localized in the nucleus and able to regulate the transcription of their parental genes through interactions with U1 small nuclear RNA (snRNA), Pol II, and promoter regions [29]. In addition, circRNAs may also be implicated in alternative splicing regulating transcription, translation, and miRNA levels [18].

#### 2.3.3. circRNAs Can Act as RNA-Binding Protein Sponges or Decoys

circRNAs can also serve as protein decoys or antagonists to influence gene expression and cellular function. In line with this, RNA-binding proteins (RBPs) harbor specific sequences to bind their specific RNA targets and regulate several cellular and molecular processes [33]. The interaction between circRNAs and RBPs was explored recently and was shown to affect the fate of their target mRNAs. For instance, circular RNA poly (A) binding protein nuclear 1 (circPABPN1) can recruit the RBP Human antigen R (HuR) to suppress its interaction with PABPN1 mRNA, which leads to reduced PABPN1 translation [33]. In vascular tissue, circular antisense noncoding RNA in the INK4 locus (*circANRIL*) sequesters pescadillo homologue 1 (PES1) which is an essential 60S-preribosomal assembly factor to impair rRNA maturation, resulting in apoptosis [34]. circFoxo3 interacts with p21 and cyclin-dependent kinase (CDK2) to form a complex that impacts cell survival and proliferation [31]. Additionally, several circRNAs have been shown to bind, store, and even insulate RBPs from specific subcellular sites [35], but the exact mechanism of dynamic interactions between circRNA transcripts and various proteins remains partially explored. Advances in this field may come from the emergence of novel high-throughput experimental technologies and innovative machine learning models for predicting RBP-binding sites on RNAs. In this context, a new computational model designated CircSLNN is now available to predict potential RBP sites in circRNA sequences [36].

#### 2.3.4. circRNAs May Encode Proteins or Peptides

Although circRNAs have been defined as a distinct class of ncRNAs that do not code for proteins, intriguingly, recent studies have indicated that some circRNAs may have an unexpected protein-coding potential [37], when recognized by ribosomes in the presence of internal ribosome entry sites (IRESs) [38,39]. Like lncRNAs, certain circRNAs may contain putative short open reading frames (ORFs) with the capacity to encode small peptides [40]. There are multiple examples in the literature of specific circRNAs that can encode peptides or proteins. For instance, circ-ZNF609 can make a protein functioning in muscle development [41]. The circβ-catenin that is derived from the β-catenin gene has been shown to encode a novel 370-amino acid β-catenin isoform [42]. Zhang et al. reported that the circular form of LINC-PINT (Long Intergenic Non-Protein Coding RNA, P53 Induced Transcript) can be translated into a small peptide to suppress glioblastoma cell proliferation [43]. However, in which condition is circRNAs translation prevalent and what role do the putative peptide/protein products play are important questions that must be asked. In addition, even though several mechanisms for circRNAs translation have been proposed [39,43], further in-depth studies are required which may help to unravel the mystery of these RNAs species. Moreover, if the translation process of circRNAs turns out to be true, this could represent additional evidence that these transcripts are functional molecules. Such a new perspective could be considered as the first step to understanding the hidden human proteome encoded by ncRNAs and highlight specific avenues for future research.

#### 2.3.5. Other Proposed Functions of circRNAs

In addition to the above-mentioned functions, it has been suggested that circRNAs found in these extracellular vesicles could serve as essential messengers for inter-cellular/inter-tissue cross-talk as released exosomes can be taken up by other cells [15,44,45]. It has also been indicated that circRNA can serve to protect mRNA from degradation. As an example, a circRNA named circPan3 appears to protect mRNAs encoding the cytokine receptor subunit IL-13Rα (IL13ral) from an mRNA decay protein, K-homology splicing regulatory protein (KSRP), and promote the production of IL-13Rα1 in crypt mouse multipotent intestinal stem cells [46]. Other studies have indicated that circRNAs could play a role in the storage, sorting, and localization of miRNAs [47]. Nevertheless, despite the significant progress toward understanding circRNAs molecular biology, a unified explanation for the function of most of these RNA species is still lacking and knowledge of their regulatory mechanisms remains rudimentary. Additionally, it is not known how circRNAs are retained in the nucleus or exported to the cytoplasm. All these limitations may slow advances to develop circRNAs as biomarkers and therapeutic tools for specific diseases.

### 2.4. circRNAs in Metabolic Diseases

Although the state of the current knowledge of circRNAs biology is at a very early stage, mounting evidence points to their role as master regulators of gene expression in many diseases including metabolic disorders. In accordance with this observation, a growing number of studies revealed the dysregulation of circRNAs in association with the pathophysiology of several diseases such as diabetes, hypertension, cardiovascular diseases (CVD), and other metabolic perturbations [48,49]. For example, the aberrant expression of certain circRNAs was associated with the development of diabetes. In islet cells, the overexpression of CDR1as significantly increased insulin mRNA level and granule secretion in β cells via CDR1as/miR-7 pathway [27]. In heart function, existing evidence showed that the heart-related circRNA (HRCR) can prevent cardiac hypertrophy and heart failure by acting as an endogenous sponge for miR-223 [50]. Furthermore, circRNA myocardial infarction-associated circular RNA (MICRA) showed prognostic significance as a biomarker for risk stratification of heart failure after myocardial infarction [51]. circZNF609, one of the abundantly expressed circRNAs in endothelial cells, was significantly upregulated upon hypoxia and high glucose exposure in vitro, as well as in patients affected by diabetes mellitus, hypertension, and coronary heart disease [52,53]. In atherosclerosis, CDKN2B-AS1 or ANRIL is perhaps one of the molecularly best-studied circRNAs. Based on previous studies, circANRIL is an antisense circRNA generated by the 9p21 locus, whose single nucleotide polymorphisms (SNPs) have been linked to genome-wide association studies (GWAS) on atherosclerotic vascular disease, as well as to type 2 diabetes mellitus (T2DM) and other diseases [34,54]. Additionally, circANRIL has been found to confer atheroprotection by controlling rRNA maturation and modulating pathways of atherogenesis [34]. There is also growing evidence that circRNAs are closely linked to non-alcoholic fatty liver disease (NAFLD), a disorder that is caused by a plethora of factors including hepatic lipid accumulation, adipose tissue and mitochondrial dysfunction, a high-fat diet, obesity, a chronic inflammatory state, insulin resistance (IR), and genetic and epigenetic factors [48,55]. Finally, although more functional circRNAs are being gradually identified and some advances have been achieved in atherosclerosis, diabetes, hypertension, and CVD, the role of circRNAs in connection with dysregulated adipogenesis and obesity remains largely elusive and needs to be explored further. The next section focuses mainly on relevant circRNA networks implicated in obesity from an epigenetic perspective.

## 3. circRNAs in Obesity

### 3.1. Obesity

Obesity and its metabolic consequences have been considered as one of the most threatening health burdens of modern times. Multiple investigations have brought forward evidence that obesity is a complex condition with multiple etiologies which develop as a joint effect of a variety of factors such as biological, genetic, social, environmental, and behavioral determinants [56,57]. The pathogenesis of overweight and obesity has been associated with altered adipose tissue metabolism and represents an important driving factor for many human metabolic disturbances and serious comorbidities including T2DM, CVD, and certain types of cancer [58,59]. In addition to its association with chronic diseases, obesity is also thought to increase the risk of developing severe forms of respiratory failure. Indeed, emerging studies revealed a strong association between obesity and the ongoing pandemic of coronavirus disease (COVID-19), which is caused by infection with severe acute respiratory syndrome coronavirus 2 (SARS-CoV-2) [60,61]. Thus, the inexorable global rise of obesity will be the toughest challenge to face and demands novel and more effective therapies.

Evidence for a large contribution of genetic variation to inter-individual differences in body mass index (BMI) comes from twin, human linkage, and association studies of large cohorts. Heritability estimates for BMI range from 31–90% across different family studies [62], leaving the remaining variance attributed to environmental factors. However, the genetic variations, measured through familial studies, affecting obesity and variations identified at different loci, together have been estimated to explain no more than 30% of the phenotypic variation [63,64]. Hence, the “missing heritability” could be attributed to many more susceptibility factors that remain to be uncovered. One of the suggested mechanisms that may account for the missing heritability is relative to epigenetic programs. Epigenetics can be defined as acquired changes in chromatin structure through cell division that arise independently of an alteration in genomic DNA sequences [65]. Epigenetic changes are dynamic and potentially reversible marks affecting gene regulation. They can include three main categories: DNA methylation, histone modifications, and ncRNAs [66]. Ongoing research is revealing the extent of the influence of epigenetics in many diseases. In support of this claim, epigenetic differences between individuals have been found to contribute to the explanation of the monozygotic twin discordance rates for common phenotypes [67]. While DNA methylation and histone modifications occur at the level of chromatin and are well-recognized as drivers for the disease phenotype, ncRNAs represent a relatively new concept in epigenetics and act mainly at the transcriptional and posttranslational levels. Concerning obesity, studies have reported that epigenetic change plays a key role in the occurrence and development of this medical condition [68]. In addition to classical epigenetic modifications, a variety of ncRNAs have been uncovered in different cells and organs including adipose tissues, many of which are involved in the regulation of adipogenesis and other metabolic processes implying their role in the etiology of obesity [69]. While lncRNAs and miRNAs are extensively investigated in obesity biology [69,70,71], studies of circRNAs in this respect have just begun.

### 3.2. circRNAs in Adipogenesis and Obesity

Despite the established link between circRNAs and several metabolic diseases, investigations on the potential connection between circRNAs and adipogenesis remain rare. Currently, there are few emerging studies extending the scope of the disease-relevant role of circRNAs to obesity and underlying mechanisms. Hence, a review of examples in the literature, suggesting key regulatory roles of circRNAs in many biological processes associated with obesity, including adipogenesis and adipocyte differentiation, is discussed next.

*Animal adipose tissue*. Emerging evidence from in vitro and in vivo animal studies suggest that circRNAs are expressed in adipose tissues and may modulate adipogenesis and lipid metabolism. In this respect, Li and colleagues identified several circRNAs differentially expressed in the subcutaneous adipose tissue of Large White pigs and Laiwu pigs [72]. A further analysis revealed that circRNA_11897 was the most significantly downregulated, whereas circRNA_26852 was the most significantly upregulated; both circRNAs were significantly involved in pathways associated with adipocyte differentiation and lipid metabolism [72] (Table 1). In another study, Liu X et al. identified 850 circRNAs differentially expressed during subcutaneous adipogenesis in Chinese Erhualian pigs. These transcripts were shown to be implicated in multiple biological processes including lipid metabolic and cell differentiation processes [73]. More recently, an interesting study indicated that circSAMD4A (sterile alpha motif domain containing 4A; also named hsa_circ_0004846) controls adipogenesis in obesity by binding to miR-138-5p [74]. In high-fat diet (HFD)-induced obese mice, mmu_circ_0000529 (the homologous mouse circRNA for circSAMD4A) knockdown reversed the associated weight gain, reduced food intake, lowered body fat, and increased energy expenditure. It is worth noting that these mice also exhibited increased insulin sensitivity and glucose tolerance [74]. Mechanistically, in vitro experiments showed that circSAMD4A can bind to miR-138-5p and act as a miRNA sponge to subsequently regulate EZH2 expression [74]. In humans, circSAMD4A was found to be significantly upregulated in obese compared to lean individuals and its level of expression notably correlated with poor prognosis in obese patients [74] (Figure 1). Functional analysis confirmed that circSAMD4A overexpression can regulate preadipocytes differentiation and effectively predict obese human outcomes. A recently published report indicated that the expression of two circRNAs, 19:45387150|45389986 and 21:6969877|69753491, was strongly correlated with fat deposition associated genes in Chinese Buffalo (*Bubalus bubalis*) [75]. In the same context, Zhang et al. identified six circRNAs, novel_circ_0009127, novel_circ_0000628, novel_circ_0011513, novel_circ_0010775, novel_circ_0006981, and novel_circ_0001494 that were related to Yac (*Bos grunniens*) adipogenesis [76]. In cattle adipocytes, circFUT10 was found to promote adipocyte proliferation and inhibit adipocyte differentiation via sponging let-7 binding of let-7c [77]. Moreover, ciRS-7/CDR1as expression levels were decreased both in ob/ob and db/db mice, which were severely obese due to lack of leptin or the leptin receptor, respectively [78]. Collectively, these findings suggest that circRNAs may participate in adipocyte differentiation and adipose tissue formation through post-transcriptional regulation.

*Human adipose tissue*. The past two years have witnessed a significant increase in the number of studies determining the function of circRNAs in human adipogenesis and obesity. In a study involving visceral adipogenesis, as many as 4080 circRNA species were found to be differentially expressed in human visceral preadipocytes (HPA-v) versus HPA-v that were induced to form adipocytes; among them, 2215 and 1865 circRNAs were significantly up- and downregulated, respectively [79]. Further validation experiments confirmed that hsa_circ_0136134, hsa_circ_0017650, and hsa-circRNA9227-1 were the most upregulated transcripts, suggesting their close association with visceral adipogenesis [79].

In order to screen circRNAs involved in adipogenesis and obesity, Arcinas and colleagues analyzed the transcriptome of human and mouse visceral and subcutaneous fat by RNA sequencing methods [80]. In this study, thousands of adipose circRNAs were identified to be regulated during adipogenesis. Among these, circTshz2-1 and circArhgap5-2 (the regulatory Rho GTPase activating protein 5-2) were revealed to be key regulators of adipogenesis in vitro [80]. Moreover, silencing of circArhgap5-2 in vivo resulted in inhibition of lipid droplet accumulation and downregulation of adipogenic markers suggesting that circArhgap5-2 might have a crucial role in maintaining the global adipocyte transcriptional program implicated in lipid biosynthesis and metabolism. Interestingly, the proadipogenic function of circArhgap5-2 was found to be conserved in human adipocytes. However, the mechanism by which circArhgap5-2 modulates adipogenesis remains to be determined. These data robustly indicate circRNAs are a contributing factor during adipogenesis and adipocyte metabolism. Another study by Schmidt et al. reported that the overexpression of H19, a maternally imprinted lncRNA that plays a role in lipid metabolism, protects against obesity, and improves insulin sensitivity [81]. In samples from patients with metabolic syndrome, a condition that is associated with abdominal obesity and CVD, the level of hsa_circH19 derived from H19 pre-RNA, was found to be highly increased and significantly correlated with variables of adiposity including body mass index (BMI), waist circumference, fat percent, high-density lipoprotein cholesterol (HDL-c), and visceral fat area [82]. Conversely, silencing of hsa_circH19 promoted human adipose-derived stem cells (hADSCs) adipogenic differentiation presumably through the interaction of such a circRNA with polypyrimidine tract-binding protein 1 (PTBP1) [82].

Another circRNA that has attracted substantial attention for its role in various physiopathological processes is circular antisense noncoding RNA at the INK4 locus (*circANRIL*). Genetic variants at the *ANRIL* gene have been linked with increased risk for T2DM, atherosclerotic CVD, coronary artery disease, myocardial infarction, and obesity [83,84,85]. Further studies have demonstrated that epigenetic regulation of *ANRIL* promoter methylation at birth is associated with increased cardiovascular risk [86] and later childhood adiposity [87]. Hence, perinatal methylation at *ANRIL* loci could be a marker for later adiposity. Collectively, the results from the above studies demonstrate that several circRNAs are differentially expressed in adipose tissue and support a significant role of these RNA species in the regulatory networks of adipogenesis. However, the precise role that circRNAs play in fat deposition and lipid metabolism remains elusive. Deeper understanding of molecular mechanisms controlling the expression of these RNAs species is critical to identify new targets for prevention of adipogenesis and therefore occurrence of obesity and obesity-related metabolic disorders.

### 3.3. circRNAs in Obesity-Induced Insulin Resistance

It is widely agreed that IR is strongly associated with obesity and T2DM, although not all individuals with obesity develop IR. Overwhelming evidence suggests that obesity-induced inflammation is characterized by the abundance of immune cells, which increase secretion of proinflammatory cytokines that act to perpetuate systemic inflammation, impair glucose tolerance, and cause IR leading to the development of T2DM [88]. Furthermore, the role of ncRNAs in the regulation of obesity and IR has been already reported [89,90]. For instance, miR-33 has been shown to play a crucial function in IR and obesity [91]. In humans, miR-122, miR-143-3p, and miR-652-3p have also been reportedly linked with obesity and IR and implicated in the modulation of genes and protein cascades in insulin signaling [92]. Furthermore, altered expression of lncRNAs has been associated with poor glycemic control, IR, accelerated cellular senescence, and inflammation in diabetes patients [93].

Up to now, no studies have specifically exploited the role of circRNAs in obesity-IR-T2DM settings, but this scenario will likely change in the future as alterations in many circRNAs in association with IR have been noticed. For instance, circHIPK3 has been shown to contribute to hyperglycemia and IR via sponging miR-192-5p and upregulating FOXO1 [94]. It noteworthy to mention that circHIPK3 has already been demonstrated to play a crucial role in diabetes retinas by virtue of its effect on miR-30a-3p [95]. circRNA-TFRC (transferrin receptor) has also been associated with IR, and the overexpression of TFRC can aggravate the risk of T2DM and metabolic diseases [96]. Even though these studies do not provide a direct link between potential circRNA signatures and obesity-related IR, they highlight this matter for further elucidation.

### 3.4. circRNAs in Adipose Inflammation

Chronic low-grade inflammation is now recognized as a hallmark of obesity and a key risk factor for IR and the development of T2DM [88,97]. Excess adiposity typically induces the recruitment of immune cells into fat depots. These immune cells, mainly macrophages, release proinflammatory cytokines/chemokines that can act locally but also systemically after being released into the circulation, therefore activating chronic inflammation which contributes to the development of obesity and associated metabolic disorders [98]. Thus, better exploring white adipose tissue inflammation mechanisms and pinpointing the immunological events occurring in this tissue will provide insights into the pathophysiological role of inflammation in obesity and help to manage obesity related diseases.

Although there has been some progress in the role of ncRNAs in adipogenesis, the significance of these RNAs in adipose inflammation remains elusive. The implication of some ncRNAs in the regulation of obesity-associated inflammation has been suggested previously. As an example, Stapleton et al. identified a novel lncRNA named macrophage inflammation-suppressing transcript (MIST) which was downregulated in both peritoneal macrophages and adipose tissue macrophages from high-fat diet-fed obese mice [99]. Moreover, human ortholog of MIST was found to be expressed in human adipose tissue macrophages and inversely correlated with obesity and IR. Recently, an elegant study by Arcinas and colleagues demonstrated a general decrease of circRNA transcripts in adipose tissue of HFD mice, suggesting that inflammation may affect circRNA biogenesis [80]. This assumption was based on the observation that treatment of differentiated primary mouse subcutaneous adipocytes with soluble tumor necrosis factor (TNF)-α led to significant reduction in circRNAs expression [80].

In another study, it was observed that circARF3 (ADP-ribosylation factor 3) functions as an endogenous miR-103 sponge to inhibit miR-103 activity, resulting in an increase of TNF receptor-associated factor 3 (TRAF3) expression and accordingly alleviates inflammation in mouse adipose tissue [100]. Subsequent experiments provided more consistent support for the notion that circARF3 is involved in inflammation as adipose inflammation was improved because TRAF3 blocked the nuclear factor κB (NF-κB)-signaling pathway, promoted mitophagy, and suppressed NOD-like receptor family, pyrin domain-containing protein 3 (NLRP3) inflammasome activation and inflammatory cytokine release [100]. On the contrary, another study reported that myeloid cell TRAF3 promotes metabolic inflammation, insulin resistance, and hepatic steatosis in obesity [101]. These authors further showed that myeloid TRAF3 may have anti-inflammatory and proinflammatory activities in lean and obese mice respectively, suggesting that, in obesity progression, myeloid TRAF3 functionally switches its activity from anti-inflammatory to proinflammatory modes.

Obesity and inflammation have been associated with several complications including T2DM, CVD, hypertension, and stroke [102]. In this perspective, Fang et al. [103] indicated that the expression of circANKRD36 was upregulated in peripheral blood leucocytes and correlated with chronic inflammation in T2DM, suggesting that circANKRD36 can be used as a potential biomarker for screening chronic inflammation in patients with T2DM. As it is estimated that a total of 80% of individuals with type 2 diabetes are obese [104], it will be interesting to evaluate the role of circANKRD36 in obesity inflammatory context.

### 3.5. Role of circRNAs in White Adipose Tissue Browning

Unlike white adipocyte tissue (WAT), which primarily stores lipids, brown adipocyte tissue (BAT) can promote energy metabolism by decreasing adiposity and increasing energy expenditure. Loss of BAT activity may contribute to obesity and development of IR. Hence, WAT browning has gained considerable attention for its potential to reverse obesity and related metabolic complications [105]. A variety of stimuli and factors such as dietary factors, cold exposure, nuclear receptors and ligands, certain drugs, and some ncRNAs can induce a phenotypic switch in adipose tissue from WAT to BAT and regulate browning [106,107,108,109]. Although significant progress has been made in understanding the epigenetic molecular mechanisms of WAT browning, the role of ncRNAs, a novel class of regulatory determinants in this context is still mostly unknown.

So far, several miRNAs have been identified and characterized to govern WAT browning process [107,110]. Moreover, a class of lncRNAs has also been shown to regulate brown adipogenesis [80,111]. With respect to the potential implication of circRNAs in such a process, studies are only emerging. In this respect, Zhang et al. reported that plasma exosomal ciRS-133 derived from gastric cancer patient cells promotes WAT browning by targeting the miR-133/PRDM16 pathway [112]. In the same manner, circNrxn2 was shown to promote WAT browning by acting as a miR-103 sponge (Table 1) and enhance the expression levels of fibroblast growth factor 10 (FGF10) in HFD mice [113]. Nonetheless, these pilot studies provide a great potential therapeutic strategy to reduce the excessive energy stores in obesity. Therefore, further investigation of the role of circRNAs in WAT browning program is needed.

## 4. Conclusions and Future Perspectives

circRNAs are increasingly being recognized to play essential roles in several diseases including metabolic disorders. They are also emerging as a novel regulatory layer in adipogenesis and lipid metabolism involved in the development of obesity. Yet a number of key questions remain: i) While the studies discussed above have confirmed that circRNAs display altered expression patterns in adipose tissue and obese individuals, the cell types and tissue origin of circRNAs in obesity are not yet fully explored. This is very challenging because obesity is a complicated condition involving many tissues and organs including the muscle, pancreas, liver, and adipose tissue. In addition, there is growing evidence that WAT is heterogeneous, and adipocytes are only part of the adipose deposit. Furthermore, different types of adipocytes have differing metabolisms and their ability to communicate with other metabolic organs through sending out various signaling molecules and possibly ncRNAs may contribute to the regulation of systemic energy homeostasis differently [114,115]. Therefore, part of the solution to these hurdles may be achieved by 1) discriminating the cellular origin of secreted circRNAs in order to identify those involved in tissue function in health and dysfunction in disease; 2) identifying potential circRNAs mediating inter-organ metabolic communication as well as those that my impact homeostasis in case of organ failure; and 3) characterizing natural circRNAs carriers to reach distant organs. ii) The potential role of exosomal circRNA signature in the development of obesity and related complications remains largely unknown. Several studies found that ncRNAs in secreted exosomes can be transferred to target tissues or cells to exert function [116]. Furthermore, adipocytes have been shown to produce and release vesicles containing genetic material to communicate with neighboring cells within WAT or facilitate metabolic organs crosstalk [117,118]. Clear and convincing evidence of this phenomenon is provided by a recent study showing that adipose tissue can release miRNAs in the circulation [115] which can regulate gene expression in other distant metabolic organs. Moreover, adipose tissue macrophages in obese mice can secrete miRNA-containing exosomes, which cause glucose intolerance and IR when administered to lean mice [116]. With respect to circRNAs found in adipose tissues, they may also be released to the circulation inside macrovesicles and have functions in target organs. In support of this assumption, a recent report indicated that adipose-derived exosomes mediate the delivery of circRNAs and promote the tumorigenesis of hepatocellular carcinoma (HCC) by regulating the deubiquitination-related miR-34a/USP7 axis [119]. As stated above, a study by Zhang et al. revealed that exosomal circRNA derived from gastric tumor promotes white adipose browning by targeting miR-133 [112]. Moreover, exosomal circRNAs have been suggested as circulating biomarkers for the diagnosis of cancer as they have been shown to discriminate patients with cancer from healthy controls [120]. However, how many copies of circRNA molecules these exosomes harbor needs to be evaluated. Further, more in vitro and in vivo modeling of exosome-mediated circRNA communication along with development of sensitive bioinformatic methods and mathematical mass-action models to capture all circRNA–target interactions, will undoubtedly provide insight into the function of these exosomal RNA species. Further stoichiometric analysis is required to better explore circRNA–miRNA and circRNA–RBP interactions. Success in addressing all these issues may clarify the controversy surrounding the sponging function of circRNAs. iii) circRNAs have also been reported to bind, store, and even insulate RBPs from specific subcellular sites or act as competitive elements to influence the function of RBPs. However, to my knowledge, no one has specifically interrogated the role of circRNA–RBP axis in adipose tissue and obesity processes. Several RBPs have been reported as adipocyte regulators by affecting different aspects of RNA processing. For example, the RNA-binding protein PSPC1 (paraspeckle component 1) has been identified as an adipogenic factor that directly interacts with adipocyte RNAs and promotes their export from the nucleus to the cytosol [121]. KH-type splicing regulatory protein (KSRP) targeted deletion has been shown to promote browning of WAT through reduction in miR-150 expression [122]. RNA-binding protein insulin growth factor 2 mRNA-binding protein 2 (IMP2) controls energy metabolism by suppressing mRNA translation of mitochondrial proton transporter uncoupling protein 1 (UCP1) and other mitochondrial mRNAs in BAT [123]. More interesting, two recently published studies reported that the RNA-binding protein HuR protects against obesity and IR [124,125]. However, it remains unclear as to whether this mechanism involves an interaction with circRNAs or not. As stated before, circRNAs containing RBP binding sites may serve as sponges or decoys for these proteins and are thus predicted to function as robust posttranscriptional regulators of gene expression. Unfortunately, the biochemical stoichiometry between circRNAs–miRNAs and circRNAs–RBPs is not yet well determined in adipose tissue. The relative abundance of circRNAs, miRNAs, and RBPs as well as their epigenetics modifications (e.g., hypermethylation) and stoichiometry analysis must be considered for the physiological relevance of any sequestration effect and cross-regulation as it may support the concept that circRNAs can serve as protein sponges or decoys to influence their cellular functions. Nevertheless, regardless of their function, the potential use of circRNAs as biomarkers for obesity and metabolic diseases remains a great promise. iv) Although the existing studies support the potential value of circRNAs in the diagnosis and therapeutics of obesity, it is too early to consider the feasibility of these molecules as biomarkers and disease therapeutic targets in a clinical obesity setting. As of now, the effectiveness of circRNAs has not been explored in large, clinically controlled, and conclusive cohorts, nor their mechanisms of action extensively studied. In addition, potential toxicity, reaction to drugs, immune response, accumulation to other tissues, and adequate carriers of circRNAs remain unknown. Nevertheless, a greater understanding of these issues is required if we are to see circRNAs clinical translation becoming a reality. v) Since obesity is a context of metabolic stress associated with dysfunction of numerous biological processes including adipose tissue dynamic, lipid metabolism, insulin signaling pathways, adipokines secretion, systemic inflammation, and mitochondrial activities, it is not clear how all these parameters might affect the machinery of circRNAs biogenesis, secretion, transfer, and mode of action. vi) Last but not least, given the complexity of obesity pathomechanisms, relying on the pattern of a single circRNA for diagnosis and treatment obesity may not be biologically most relevant and sufficiently specific to provide a clinical utility. Rather, one should consider a multi-markers approach that combines candidate circRNAs/signatures, miRNAs, and RBPs which may be highly discriminative, accurate, and efficient in predicting obesity and associated metabolic perturbations.

To sum up, circRNAs have recently emerged as a class of ncRNAs with multifaceted roles in the cell. Emerging evidence from in vitro and in vivo experimental studies indicates that circRNAs are involved in the regulation of adipogenesis and obesity. However, the ongoing efforts to better understand the role of circRNAs in adipose tissue and metabolics must include the above-mentioned considerations as they may help accelerate their clinical research.

## Figures and Tables

**Table 1:** Relevant circular RNAs involved in adipogenesis and obesity-related metabolic complications.

circRNA	Cell/Tissue Type	Expression	Potential Function	References
circRNA_11897	Pig subcutaneous adipose tissue	↓	Involved in the regulation of adipogenic differentiation and lipid metabolism	[72]
circRNA_26852	Pig subcutaneous adipose tissue	↑	Regulates adipogenic differentiation and lipid metabolism	[72]
circSAMD4A (hsa_circ_0004846)	VAT from obese patients	↑	Overexpression of circSAMD4A potentially regulates preadipocytes differentiation and correlates with a poor prognosis in obese patients	[74]
circFUT10	Bovine adipose tissue	↑	Plays a role in adipocyte proliferation and inhibits adipocyte differentiation via sponging let-7	[77]
hsa_circ_0136134, hsa_circ_0017650	HPA-v/adipocytes	↑	May influence HPA-v differentiation by regulating their parental genes expression, *LPL* and *ITIH5, respectively*	[79]
hsa-circRNA9227-1	HPA-v/adipocytes	↑	Regulates adipogenesis by recruiting hsa-miR-665	[79]
circTshz2-1, circArhgap5-2	Human/mouse visceral and subcutaneous adipose tissues	↑	Implicated in the regulation of adipogenesis, adipocyte metabolism, and obesity	[80]
hsa_circH19	Human blood sample	↑	Significantly correlates with BMI, waist circumference, and visceral fat in serum of metabolic syndrome patients	[82]
circARF3 (circ0000650)	Mouse adipose tissue	↑	Acts as an endogenous miR-103 sponge to alleviate adipose inflammation by promoting mitophagy	[100]
circNrxn2 (circ005661)	Mouse adipose tissue	-	Promotes WAT browning and mitochondria functions	[113]

Abbreviations: circSAMD4A, sterile alpha motif domain containing 4A; BMI, body mass index; HPA-v, human preadipocytes from visceral fat tissue; ITIH5, inter-alpha-trypsin inhibitor heavy chain; LPL, lipoprotein lipase; VAT, visceral adipose tissue; WAT, white adipose tissue.
